# Unusual Case of Osteomyelitis and Discitis in a Drug User with a Background of Chronic Back Pain: Do Not Miss the Serious Etiologies

**DOI:** 10.1155/2013/729812

**Published:** 2013-10-02

**Authors:** Alaa M. Ali, Moona Khan, Shawn G. Kwatra, Aram Barbaryan, Nasir Hussain, Raya Saba, Aibek E. Mirrakhimov

**Affiliations:** Saint Joseph Hospital, Department of Internal Medicine, 2900 N. Lake Shore Drive, Chicago, IL 60657, USA

## Abstract

Chronic back pain is a common presenting complaint that is frequently encountered by clinicians. The challenge for clinicians is identifying the relatively few patients with a significant probability of a more serious problem that requires further evaluation. Such individuals require further evaluation for possible occult malignancy, infection, or fracture. We present a case of a 50-year-old male with a past medical history of chronic back pain and IV drug abuse who presented with acute back pain and in whom a diagnosis of vertebral osteomyelitis was missed during multiple visits to the emergency room.

## 1. Introduction

Back pain ranks second only to upper respiratory illness as a symptomatic reason for office visits to physicians. An etiologic diagnosis is not established for most patients with back pain in whom episodes of back pain are self-limited and resolve without specific therapy. Diagnostic tests are only indicated if the test will change the management strategies and improve the outcomes. Otherwise, these tests, if performed in low-pretest probability population, will lead to unnecessary further workup or interventions. Nevertheless, physicians should be able to identify the cases of back pain in which further workup might reveal a serious pathology.

## 2. Case Presentation

A 50-year-old male with a history of intravenous (IV) drug abuse presented to the hospital with acute onset back pain. The patient had a 10-year history of chronic back pain as well as a history of cervical radiculopathy, for which he underwent anterior cervical discectomy and fusion 6 years ago. The patient stated that his back pain had been worsening over the past 3 weeks. The patient presented to the emergency room several times in the last couple of weeks prior to admission. His back pain was presumed to be related to his previous history, and he was discharged home on opioid analgesics.

On admission, the patient reported severe squeezing back pain extending from his lower to middle back. The pain was described by the patient as a 10/10 in intensity with radiation to his buttocks. The pain was not relieved by lying down and was aggravated by activity. The patient did not exhibit any lower extremity weakness, numbness, or bladder/bowel dysfunction, and he denied any fevers or night sweats. On physical examination, palpation of the spine revealed diffuse tenderness. Straight leg raising test was negative.

**Figure 1:**
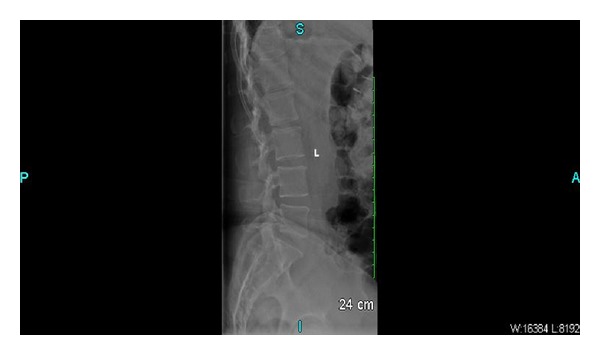
Plain lumbosacral X-ray showing degenerative changes.

In the ED plain X-ray of the lumbosacral spine revealed degenerative changes of the lumbosacral spine but no evidence of infection, malignancy, or fracture (see Figure 1). The patient was sent to the inpatient ward for observation with an initial diagnosis of chronic back pain.

**Figure 2:**
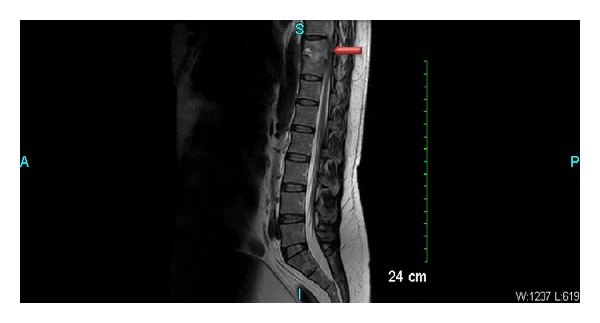
MRI of lumbosacral spine showing discitis at T10-T11 level (red arrow).

Initial laboratory findings revealed a mildly elevated white blood cell count at 12,000 and anemia with hemoglobin at 9.5. Erythrocyte sedimentation rate was found to be 92. With these laboratory abnormalities and persistent symptoms, magnetic resonance imaging (MRI) was done to further evaluate the back pain. MRI examination of the lumbosacral spine revealed osteomyelitis with discitis at T10-T11 level with some retropulsion of both vertebral bodies causing mass effect on the spinal cord (see Figure 2). The patient was immediately started on IV dexamethasone, and he underwent CT-guided aspiration of the intervertebral disc space the following day. Following the procedure, the patient was started on cefepime and vancomycin while awaiting culture results from the aspiration.

Blood cultures as well as cultures of the disc aspiration material grew *Enterobacter aerogenes*. Vancomycin and cefepime were substituted with ertapenem, because of antibiotic sensitivity results.

The patient's clinical condition started to improve on day 5 of admission, and he was discharged with long-term antibiotics therapy.

Patient was followed up 8 weeks later with resolution of his back symptoms.

## 3. Discussion

Back pain is the second most common symptom-related reason for clinician visits in the United States [1]. Up to 85 percent of adults have low back pain at some point in their lives [1, 2]. The spectrum of the etiologies for back pain is broad. For most patients with low back pain (up to 85%), a precise pathoanatomical diagnosis is often impossible, which leads to various imprecise diagnoses (e.g., sprain or strain). The vast majority of patients will have “mechanical” or nonspecific back pain [3]. Episodes of back pain are usually self-limited and resolve with symptomatic treatment.

Rarely, in less than 5 percent of the cases, acute back pain is a harbinger of serious medical illness, including infection, malignancy, or other systemic disease. However, clinicians often face significant diagnostic challenges in identifying which patients require further evaluation. Indeed, many experts believe that back pain has been “overmedicalized,” [4] and that there is evidence of excessive imaging and surgery for low back pain in the United States [5]. Cases that have clues suggesting serious conditions should be investigated and worked up even with a background of chronic back pain for years. With this clinical dilemma in mind, the American College of Radiology has published a list of ten findings, based on a patient's history, suggesting a need for radiologic investigation to evaluate potential complicated low back pain. These criteria have been referred to as “red flags” and are listed in Table 1 [6].

In a study that was performed in a public hospital, the authors evaluated 1,975 patients with a chief complaint of back pain. Thirteen patients proved to have underlying cancer. Findings significantly associated with underlying cancer included the following: age greater than or equal to 50 years, previous history of cancer, duration of pain greater than 1 month, failure to improve with conservative therapy, elevated erythrocyte sedimentation rate (ESR), and anemia [7].

Features in the history of patients with acute low back pain that indicate an increased risk for vertebral fractures and suggest further workup include the following: recent significant trauma, recent mild trauma in a patient over age 50, history of prolonged glucocorticoid use, osteoporosis, patient over age 70 [8].

Features that suggest infections include pain that is not relieved by lying down, injection drug use, skin infection, urinary tract infection, or recent fever [9–11].

In the case presented above, the patient had several red flags that were overlooked during his multiple visits to the emergency rooms by his chronic history of back pain. Those red flags included the following: history of intravenous drug use, failure to improve with conservative therapy, the severe nighttime pain, and no relief of the pain upon lying down.

The most common cause of vertebral osteomyelitis in IV drug users is *Staphylococcus aureus*, although other organisms including *Pseudomonas aeruginosa* and *Candida* can be also seen. More rarely, enteric gram-negative bacilli can also be a cause of vertebral osteomyelitis. However, these organisms typically cause spinal infection after urinary tract infection or instrumentation [12–14].

Finally it is important to keep in mind that patients with gram-positive osteomyelitis and gram-positive bacteremia should have transthoracic echocardiography to exclude the presence of bacterial endocarditis.

## 4. Learning Points

- Mechanical nonspecific chronic back pain is an exceedingly common reason for clinician visits in the US. The precise etiology for many of these cases remains unknown.
- The challenge for clinicians is to identify the relatively few patients presenting with back pain that exhibited flags indicating that they require further evaluation for possible occult malignancy, infection, or fracture.
- Although *Staphylococcus aureus* is the most important infecting organism of osteomyelitis in drug users, other organisms including *Pseudomonas aeruginosa*, *Candida* spp., or even gram-negative bacilli can be the offending agents.

## Figures and Tables

**Table 1:** “Red flags” for a potentially serious underlying cause for low back pain.

Recent significant trauma or milder trauma age > 50
Osteoporosis, prolonged use of glucocorticoids
Intravenous (IV) drug use
Unexplained weight loss
History of cancer
Immunosuppression
Duration greater than 6 weeks
Focal neurologic deficit progressive or disabling symptoms
Unexplained fever
Age > 70
